# Electron-tracking Compton camera imaging of technetium-95m

**DOI:** 10.1371/journal.pone.0208909

**Published:** 2018-12-10

**Authors:** Yuichi Hatsukawa, Takehito Hayakawa, Kazuaki Tsukada, Kazuyuki Hashimoto, Tetsuya Sato, Masato Asai, Atsushi Toyoshima, Toru Tanimori, Shinya Sonoda, Shigeto Kabuki, Hiroyuki Kimura, Atsushi Takada, Tetsuya Mizumoto, Seiya Takaki

**Affiliations:** 1 National Institutes for Quantum and Radiological Science and Technology (QST), Tokai, Ibaraki, Japan; 2 Japan Atomic Energy Agency, Tokai, Ibaraki, Japan; 3 Kyoto University, Kyoto, Japan; 4 Tokai University, Kanagawa, Japan; 5 Kyoto Pharmaceutical University, Kyoto, Japan; Emory University School of Medicine, UNITED STATES

## Abstract

Imaging was conducted using an electron tracking-Compton camera (ETCC), which measures γ-rays with energies in the range of 200–900 keV from ^95m^Tc. ^95m^Tc was produced by the ^95^Mo(p, n)^95m^Tc reaction on a ^95^Mo-enriched target. A method for recycling ^95^Mo-enriched molybdenum trioxide was employed, and the recycled yield of ^95^Mo was 70%-90%. Images were obtained with the gate of three energies. The results showed that the spatial resolution increases with increasing γ-ray energy, and suggested that the ETCC with high-energy γ-ray emitters such as ^95m^Tc is useful for the medical imaging of deep tissue and organs in the human body.

## Introduction

Technetium-99m is the most widely adopted radioisotope for medical diagnostic scans such as single-photon emission computed tomography (SPECT) [1, 2]. There are over 31 commonly used radiopharmaceuticals based on ^99m^Tc for diagnostic imaging and functional studies of the human body. Various ^99m^Tc-labeled compounds are injected into the patient’s body as radioactive tracers, and subsequently, γ-rays emitted from the accumulated ^99m^Tc are measured using position-sensitive γ-ray detectors. The half-life of ^99m^Tc (6 h) is suitable for handling in hospitals and for use in short time scans. One of the notable features of ^99m^Tc is that it emits a γ-ray with an energy of 141 keV. Because photon absorption through the photoelectric effect at this energy dominates in the interaction between photons and materials, it is possible to determine the incident direction of the γ-rays by collimation with high-Z materials. Multi-segmented scintillation detector arrays coupled with heavy metal pine-hole collimators are generally used in SPECT. However, the use of a collimator decreases the detection efficiency of γ-rays from ^99m^Tc, and requires flames to support the heavy γ-ray detector system.

If one uses a medical isotope that emits γ-rays with energies in the range of 200 keV to 2 MeV, it is possible to image deep positions in the patient’s body with relatively high spatial resolution. If a Tc isotope that emits high-energy γ rays is used as an alternative to ^99m^Tc, all radiopharmaceuticals developed for ^99m^Tc can be, in principle, used because the Tc chemistry is the same. Therefore, ^95g^Tc, ^95m^Tc, and ^96^Tc are potential candidates for high-energy γ-ray emitters. Hayakawa et al. have quantitatively estimated the relative γ-ray intensities and production rates using a compact medical cyclotron for various Tc isotopes [3]. Although the relative γ-ray intensity of ^95m^Tc is lower than that of ^99m^Tc [3], its relatively long half-life of 61 d enables its use to study the behavior of Tc isotopes in the human body. In 1976, the biological half-life of Tc was measured using ^95m^Tc instead of ^99m^Tc [4]. However, in the MeV energy region, Compton scattering is the dominant process in the interactions between γ-rays and materials; thus, it is difficult to effectively obtain clear images using the conventional SPECT detector system.

Over the last decades, the Compton camera, which was originally developed to observe stellar gamma-ray bursts [5, 6], was applied to medical uses such as medical diagnostic scanning or the monitoring of radiation therapy [7–17]. Even if the γ-ray energy is in the MeV region, one can measure γ-ray images using the Compton camera. The Compton camera provides an additional advantage in that the size and weight of the detection device system can be reduced due to the large viewing angle of the camera and absence of a collimator. To determine the direction of an incident γ-ray, both the angles of the scattered γ-ray and scattered electron should be measured. However, conventional Compton cameras can only measure the angle of the scattered γ-ray. Recently, the electron-tracking Compton camera (ETCC) was developed for astronomical observations [18, 19], the measurement of radioactivity in fields [20, 21], and medical scans [22–25].

Tanimori et al. [18–21, 23] have developed the ETCC based on a gaseous time-projection chamber (TPC). This type of ETCC consists of two parts: a TPC coupled with a micro pattern gas detector to precisely measure the track of the electron recoiled by Compton scattering on materials inside the TPC, and a position-sensitive scintillation camera to detect the scattered photon. Thus, the ETCC can measure the angles of both the scattered γ-ray and electron, thereby obtaining the direction of the incident γ-ray.

Hatsukawa et al. have first demonstrated an ETCC imaging of ^95m^Tc [24]. The 204-keV γ-ray, which is the most intense γ-ray from ^95m^Tc, was measured by an ETCC, and an image of the isotope distribution was reconstructed by employing the 204 keV γ-ray emitted. However, because the cross section of the photoelectric absorption at the energy of 204 keV is relatively strong, a part of incident γ-rays are absorbed inside TPCs. In this case, Compton camera cannot work. The energy of the final photon in Compton scattering is not high, the detection efficiency of scintillation detectors is relatively low. Thus, the previous experiment [24] was not optimized for ETCC imaging. Gamma-rays with energies as high as 582 and 835 keV are also radiated from ^95m^Tc. At these energies, Compton scattering dominates, and the energies of scattered photons are higher than those at 204 keV. Thus, clearer images are expected to be obtained than that at 204 keV.

A point for consideration in the economical production of ^95m^Tc radiotracers is the recycling of the enriched ^95^Mo targets after the chemical separation of ^95m^Tc. The chemistry for the separation of ^95^Tc from a large excess of ^95^Mo isotopes is the same as the method developed for recycling ^100^Mo in ^100^Mo(p, 2n)^99m^Tc production [26, 27]. Thus, it is expected that the previously developed recycling method is applied to ^95m^Tc/^95^Mo.

In this paper, three γ-rays with energies of 204, 582, and 835 keV from a γ-ray source of ^95m^Tc, which was produced by the (p, n) reaction, were measured using an ETCC system, and the image of the γ-ray source was reconstructed for each energy to study the energy dependence of the spatial resolution. Additionally, we demonstrated a recovery method for the enriched Mo isotope irradiation target.

## Materials and methods

### Beam irradiation and chemical separation

**Fig 1:**
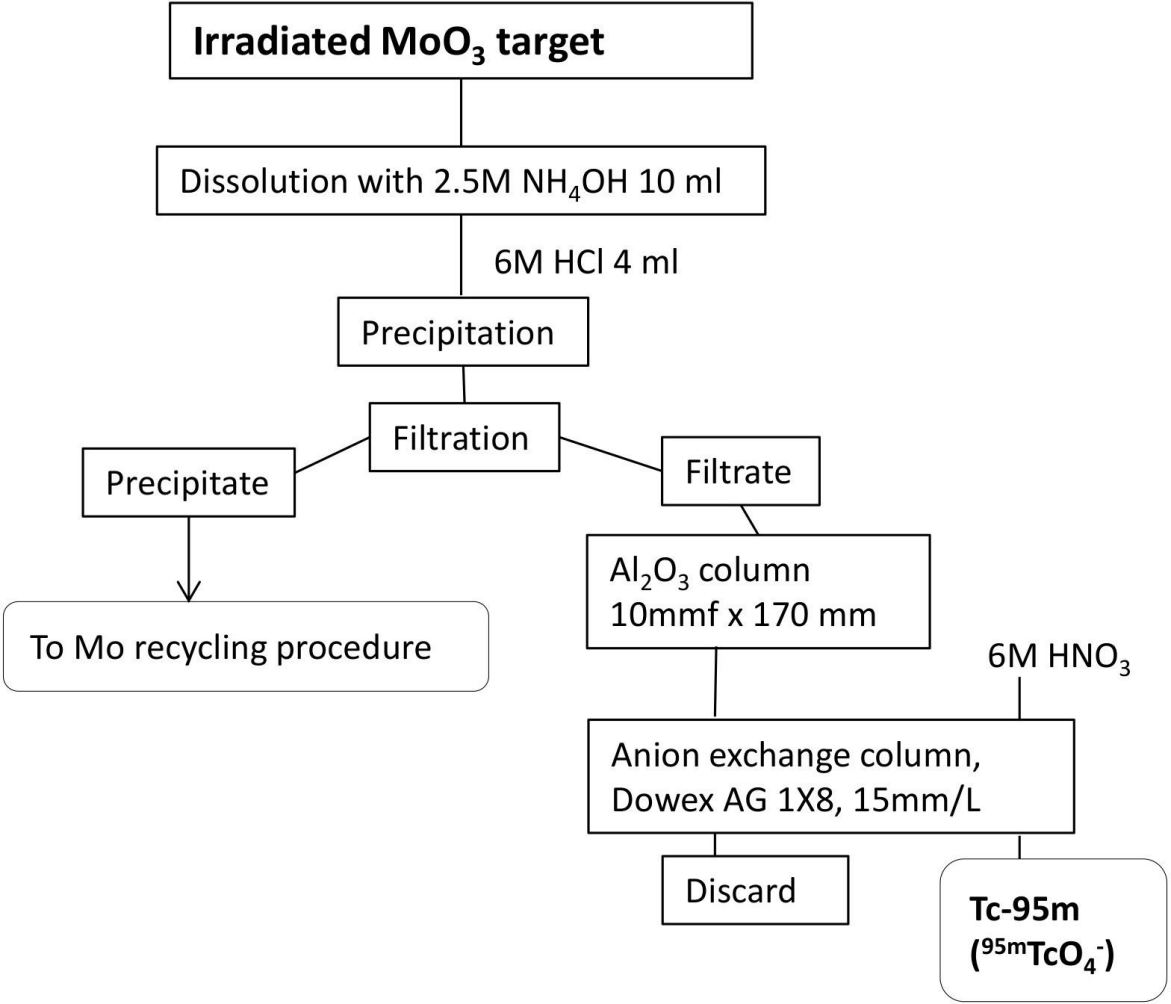
Chemical separation procedure of ^95m^Tc from the irradiated ^95^MoO_3_ target.

The experiment was carried out at the tandem accelerator facility at the Japan Atomic Energy Agency (JAEA). The target was made of ^95^Mo-enriched molybdenum trioxide powder (^95^MoO_3_ 94.80% isotopic enrichment; ISOFLEX, San Francisco, USA) with a weight of 100 mg. This enriched powder was placed in a cylinder case with a diameter of 10 mm and was pressed at 65 MPa for 30 min. The pressed ^95^MoO_3_ tablet was wrapped with high-purity aluminum foil and was used as an irradiation target. The proton beam with an energy of 15 MeV was provided from the tandem accelerator. The averaged beam current measured with a Faraday cup was typically 1.2 μA. After six weeks of cooling time, ^95m^Tc was extracted from the irradiated ^95^MoO_3_ target via the chemical procedure shown in Fig 1. The irradiated target was dissolved in 10 ml of 2.5 mol/l NH_3_OH. By adding 4 ml of 6N HCl, an insoluble precipitate was appeared. The molybdenum oxide precipitate was isolated by filtration, and the solution containing the Tc ions was purified by an Al_2_O_3_ column and an anion exchange column [24]. ^95m^Tc with a radioactivity of 500 kBq was obtained in solution.

### Recycling enriched MoO_3_ target material

**Fig 2:**
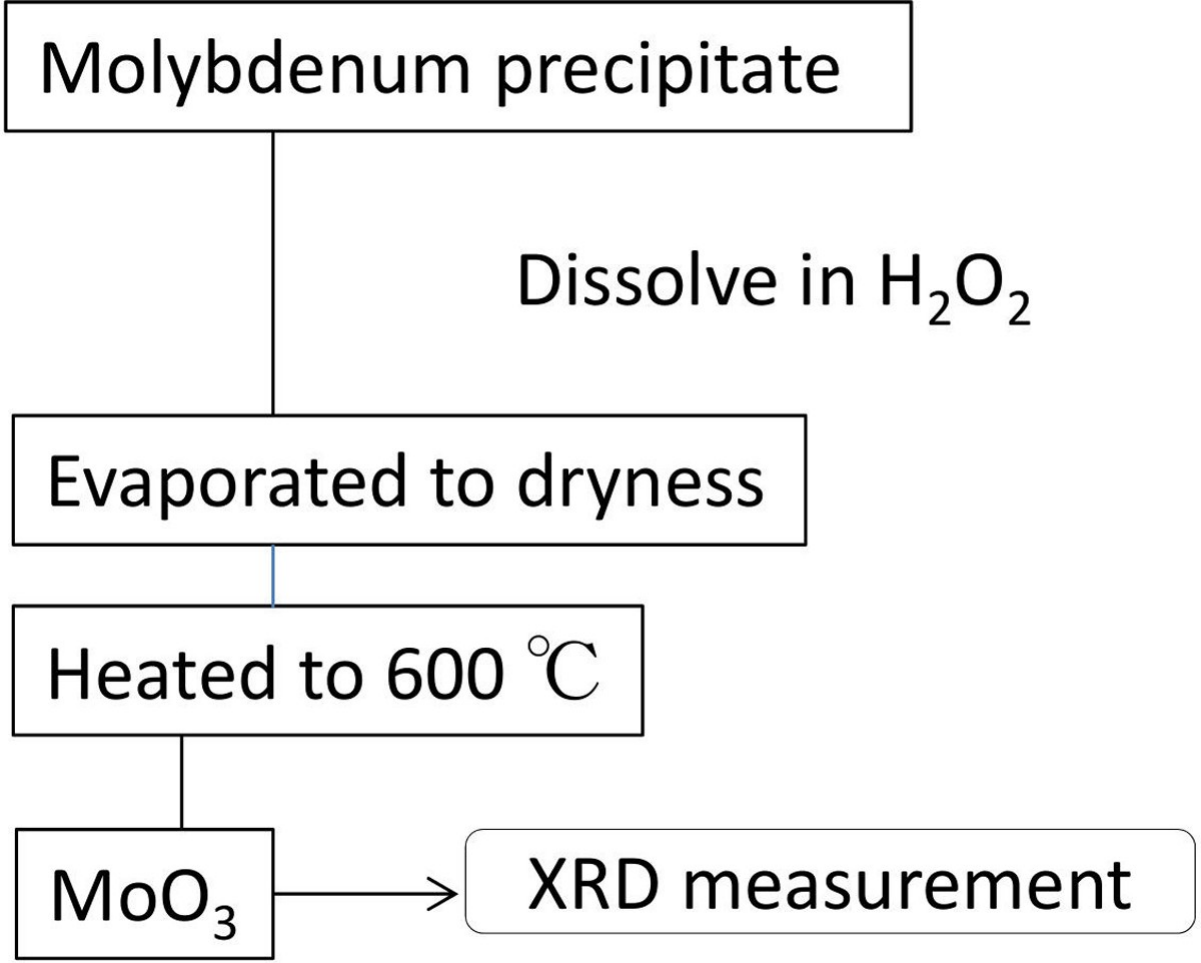
Block diagram of the recycling procedure of ^95^MoO_3_ from molybdenum oxide obtained from the ^95m^Tc separation procedure.

The molybdenum oxide precipitate, which was separated from the Tc ions, could be recycled. To repeatedly utilize precious enriched isotopes, a recycling procedure method of the enriched molybdenum isotope sample was developed. A method was previously developed for the recovery of ^100^MoO_3_ from molybdenum oxide deposits obtained from ^99m^Tc preparation [28]. This method was applied to the recycling of ^95^MoO_3_ isotope-enriched target materials. Fig 2 shows the chemical procedure used in the present study. The enriched Mo ions separated from the Tc ions were collected as a molybdenum oxide deposit, which was subsequently dissolved in 20% H_2_O_2_ solution and evaporated to dryness. The remaining Mo powder was heated to 600°C for 30 min in a muffle furnace.

### X-ray diffraction analysis

The Mo material obtained from the recycling procedure was examined by powder X-ray diffraction (XRD) analysis. XRD experiments were performed with a D8 ADVANCE diffractometer (Bruker AXS corp.) using Cu Kα radiation in the diffraction angle range 2θ = 25° to 150°. Molybdenum trioxide (MoO_3_; 99.5% Merck, Darmstadt, Germany) was used as a reference material. The obtained Mo powder and reference molybdenum trioxide were pulverized in an agate mortar. Both pulverized materials were analyzed by the XRD measurements.

### γ-ray imaging

**Fig 3:**
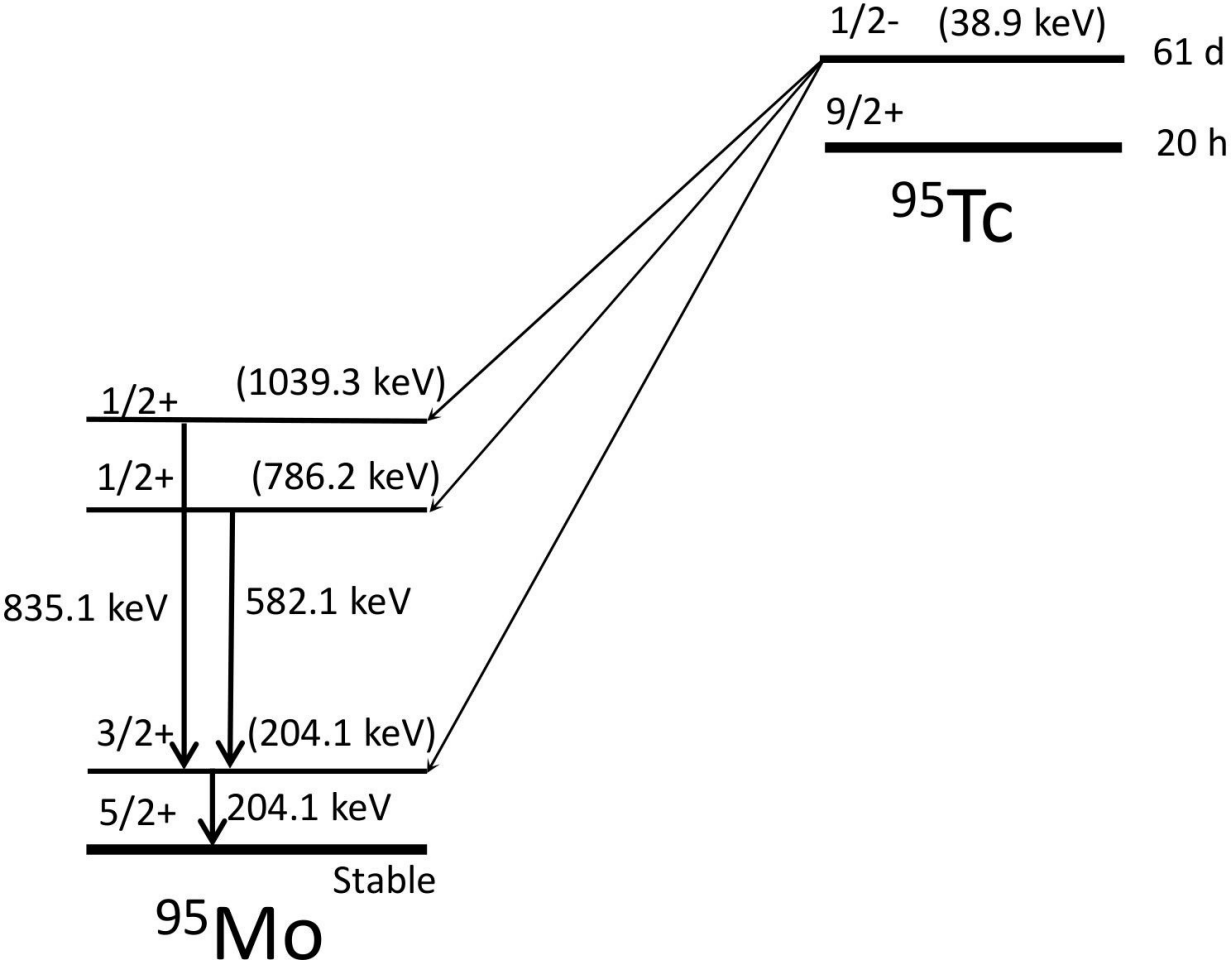
Decay scheme of ^95m^Tc. Three gamma rays, 204.1 keV, 582.1 keV and 835.1 keV, are emitted from ^95m^Tc. The gamma energies are shown by traditional arrows, and the level energies from the ground state are shown in parentheses.

The γ-rays from ^95m^Tc were measured using an ETCC system [29]. ^95m^Tc contained in a ϕ10 mm × 50 mm plastic vial was used as a radiation source. The radioactivity of ^95m^Tc was 170 kBq. This source was placed at a 48.4-cm distance from the top of the ETCC. As shown in Fig 3, there are two possible gamma-ray emitters of an isomer with a half-life of approximately 61 d and the ground state with a half-life of 20.4 h. However, the unstable ground states decay over 6 weeks of cooling time. The isomer in ^95m^Tc partly decays to excited states in the daughter nucleus, ^95^Mo by electron capture; subsequently, the excited states decay to the ground state of ^95^Mo by emission of γ-rays. In this decay, γ-rays with energies of 204.1, 582.5, and 835.1 keV are emitted. These values are within the wide energy range of the ETCC, which is 200–1300 keV.

**Fig 4:**
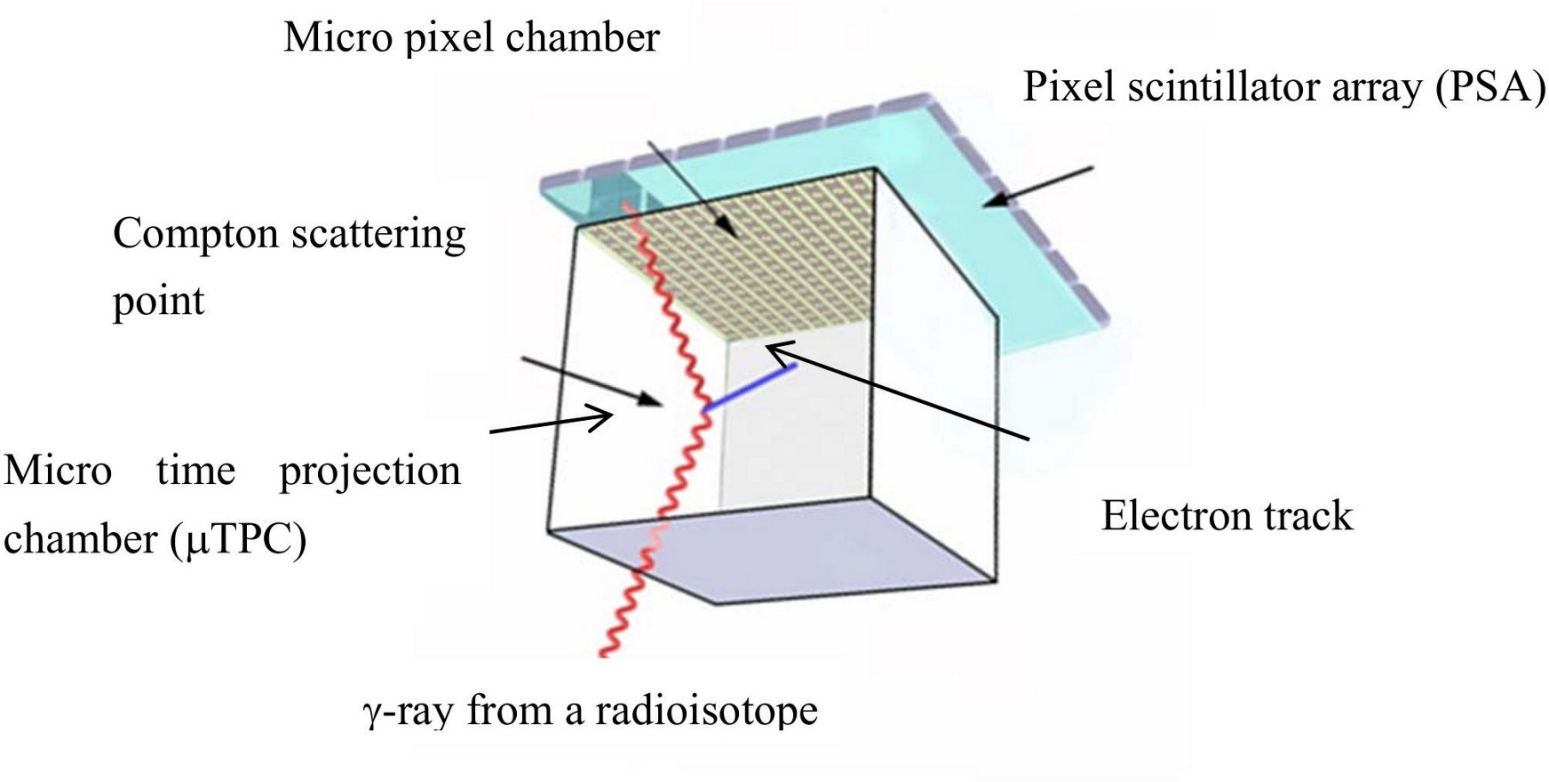
Schematic diagram of the electron-tracking Compton camera (ETCC). The detector comprises a micro-TPC and a scintillation array module. The micro-TPC (timing projection chamber) which is made from a micro pixel chamber, detects the recoil electron tracks, and the scintillator array detects the scattered gamma-rays.

The ETCC [29] consists of two detector parts, as shown in Fig 4. A γ-ray from ^95m^Tc is scattered in the first detector, which is a micro gaseous time projection chamber (μ-TPC) with a volume of 10 × 10 × 10 cm^3^ filled with a gas mixture of 90% Ar and 10% C_2_H_6_ in mass ratio and sealed at 1 atm. The readout of this μ-TPC consists of a gas electron multiplier and a micro-pixel chamber (μ-PIC). The μ-TPC can measure the 3-dimensional recoil electron track. The scattered γ-ray is measured by the second detector, which is composed of 9 pixel scintillator arrays (PSA). One PSA consists of 8 × 8 Gd_2_SiO_5_:Ce scintillator pixels with a pixel size of 6 × 6 × 13 mm^3^. Multi-anode photomultiplier tubes (Hamamatsu Photonics, Flat-Panel H8500) are used as photon sensors of the scintillator pixels; a tube consists of 8×8 anode pixels with a size of 6×6 mm^2^. The energy resolutions of the ETCC are 20 ± 2% in Full Width at Half Maximum (FWHM) at 366 keV, 15 ± 1% at 511 keV, and 11 ± 2% at 835 keV. The angular resolution is defined by two parameters the angular resolution measure (ARM) and scatter plane deviation (SPD). The shape of the image is made by a combination of the resolution of the Compton scattering angle (ARM) and directional angular resolution of the Compton scatter plane (SPD). The ARMs are 8.82 ± 0.14 deg, 6.14 ± 0.19 deg, and 5.13 ± 0.18 deg at 366, 511, and 835 keV, respectively. The SPDs are 93.5 ± 0.6 deg, 97.9 ± 1.2 deg, and 105.1 ± 1.7 deg at 366, 511, and 835 keV, respectively. These parameters are listed in Table 1.

**Table 1:** Characteristics of the ETCC used in this experiment.

γ-ray energy	energy resolution	ARM^*^	SPD^**^
366 keV	20 + 2%	8.82 + 0.14 deg	93.5 + 0.6 deg
511 keV	15 + 1%	6.14 + 0.19 deg	97.9 + 1.2 deg
835 keV	11 + 2%	5.13 + 0.18 deg	105.1 + 1.7 deg

* ARM: Angular resolution measure.

**SPD; Scatter plane deviation.

The ETCC can determine the γ-ray direction, event by event, from the electron track as well as the detection position of the scattered γ-rays and their energies. The μ-TPC can measure both the energy deposited by Compton scattering inside the μ-TPC and length of the recoiled electron track. The length of the recoiled electron track depends on its initial energy, namely, the deposited energy by Compton scattering. If a scattered electron escapes from the μ-TPC, we cannot know the energy deposited. Thus, we selected events in the case that the electron stopped in the μ-TPC from the length and deposited energy. In this experiment, approximately 13% of the total events were accepted. The direction of an incident γ-ray is determined, event by event, from the energies and directions of the scattered electron and the scattered γ-ray by the kinematics of Compton scattering. The energy of the incident γ-ray is obtained by summing the energies of the scattered γ-ray and electron. A γ-ray image is constructed with a gate of the incident γ-ray energy. We use the list-mode maximum-likelihood expectation-maximization (list-mode ML-EM) method, which is commonly employed in medical algorithm [30]. The detailed analysis method is described in previous papers [20, 29, 31].

## Results and discussion

### Beam irradiation and chemical separation

Approximately 1.2 MBq of ^95m^Tc is produced by irradiating a 15-MeV proton beam onto 100 mg of the ^95^MoO_3_ target for 7 h. Although the beam energy is higher than the threshold energy of the ^95^Mo(p, 2n)^94^Tc reaction, the abundance of ^94^Tc is much lower than that of ^95m^Tc. The irradiated MoO_3_ target is dissolved in 2.5 M NH_4_OH solution after 6 weeks of cooling time in which the radioactivity of ^95m^Tc decreases to 830 kBq. Finally, approximately 500 kBq of ^95m^Tc is obtained after chemical separation. In this separation scheme, a chemical yield of approximately 60% is obtained. Note that contaminated Tc isotopes, which are produced by (p, n) reactions on Mo isotopes (except for ^95^Mo), decay almost completely during the cooling time.

### Recycling enriched MoO_3_ target material and XRD analysis

**Fig 5:**
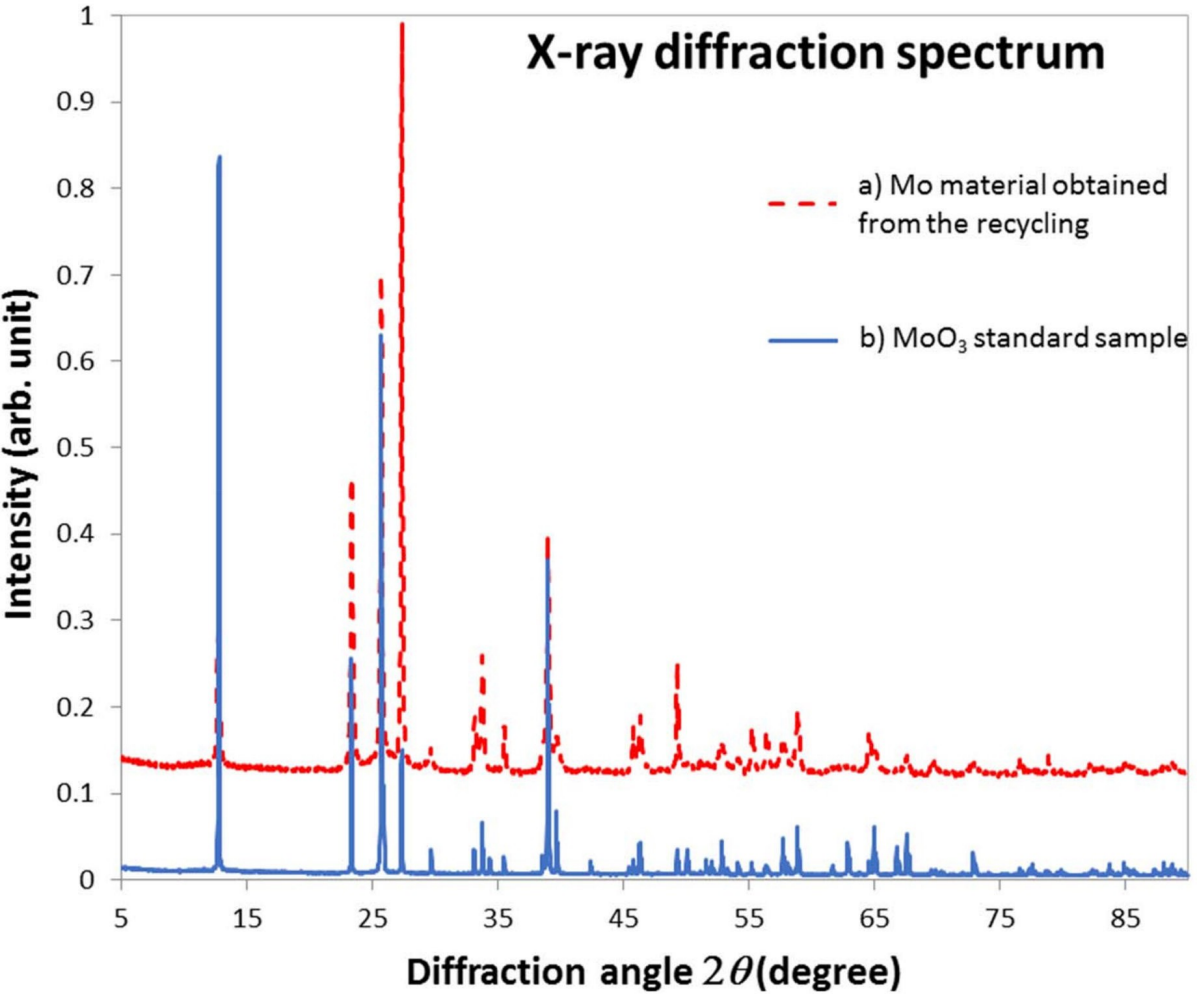
Powdered XRD spectra obtained from (a) recycled Mo oxide material (upper; dashed line) and (b)the MoO_3_ standard (lower; solid line). Both samples show the same reflect angles, indicating the recycled Mo material is MoO_3_.

The recovery of MoO_3_ (see Fig 2) was repeated three times, and recovery yields of 70%-90% were obtained. The powder XRD spectra of the recovered Mo compound (a) and standard MoO_3_ sample as the reference (b) are shown in Fig 5. These two spectra are very similar in that the positions of strong peaks that correspond to the reflection angles are almost identical. The differences in the XRD peak intensities may arise from the small difference between the crystal orientations of the sample powders. According to this result, we concluded that the two compounds (a) and (b) are identical.

### γ-ray imaging

**Fig 6:**
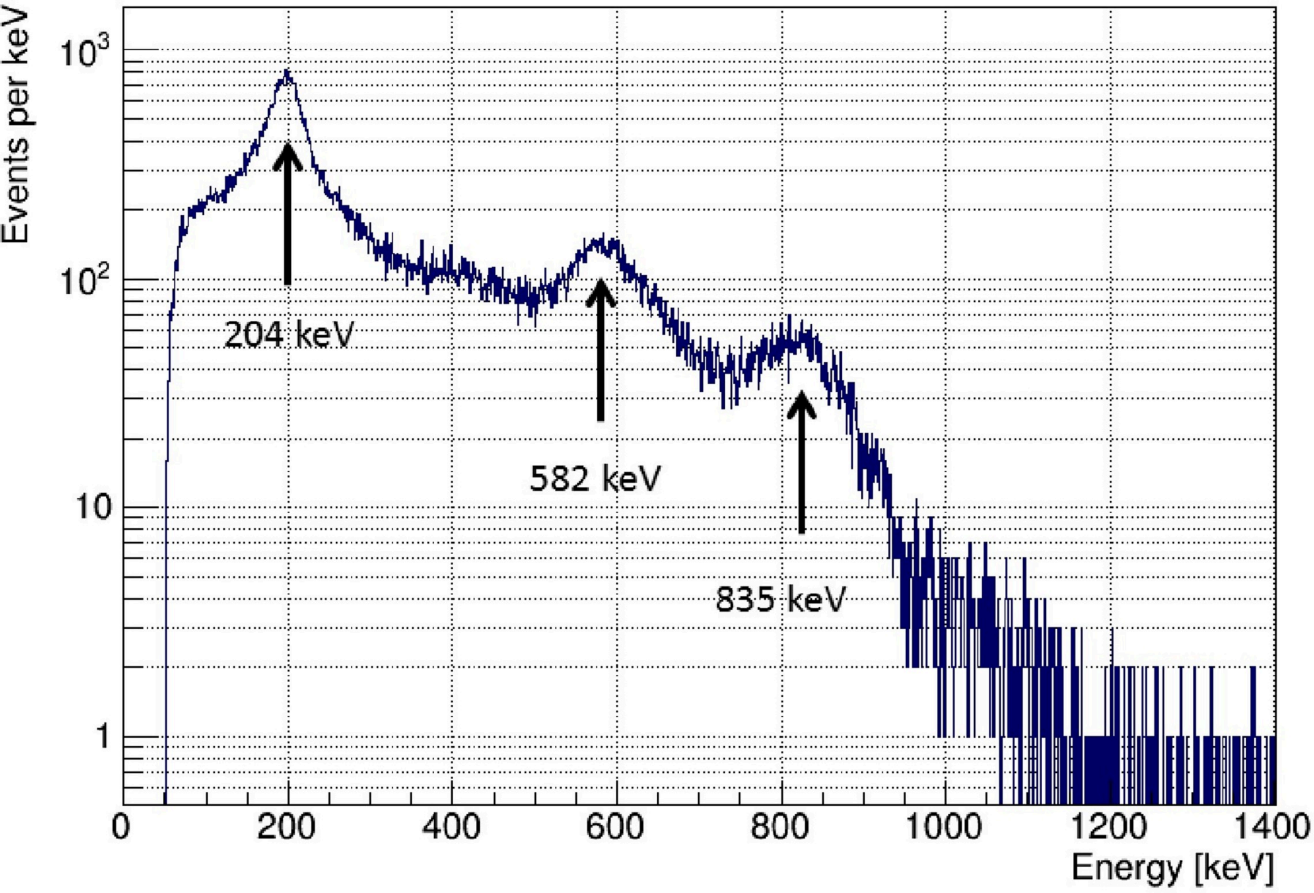
Energy spectrum of ETCC obtained from ^95m^Tc measurement. The horizontal axis shows the combination of energies of the electron and scattered gamma rays. Gated energy windows were set at 204.0±20.4 keV, 582.0±58.2 keV, and 835.0±83.5 keV.

γ-rays with energies of 204 keV, 582 keV, and 835 keV are predominantly emitted from ^95m^Tc. Using the ETCC, we obtained two-dimensional images of the ^95m^Tc source. The total number of events obtained in this measurement was 1.025 × 10^6^ events, whereas the number of events accepted was 1.299 × 10^5^. Approximately 13% of the total events were accepted. Fig 6 shows a summed energy spectrum of the energy deposited in a gaseous TPC and energy measured by a scintillator detector for each Compton scattering. When both the TPC and the scintillator measured simultaneously an electron (or a photon), we recorded it. The horizontal axis represents the total energy of the recoiled electron and scattered γ-ray. The total energy is, in principle, identical to the incident photon energy. The three peaks that appear in the spectrum correspond to the three γ-ray energies from ^95m^Tc. The energy windows of the ETCC were set at (a) 204 ± 20.4 keV, (b) 582 ± 58.2 keV, and (c) 835 ± 83.5 keV. Two-dimensional images with the gate of these γ energies were reconstructed by an iterative reconstruction technique, the ML-EM method, using list-mode data.

**Fig 7:**
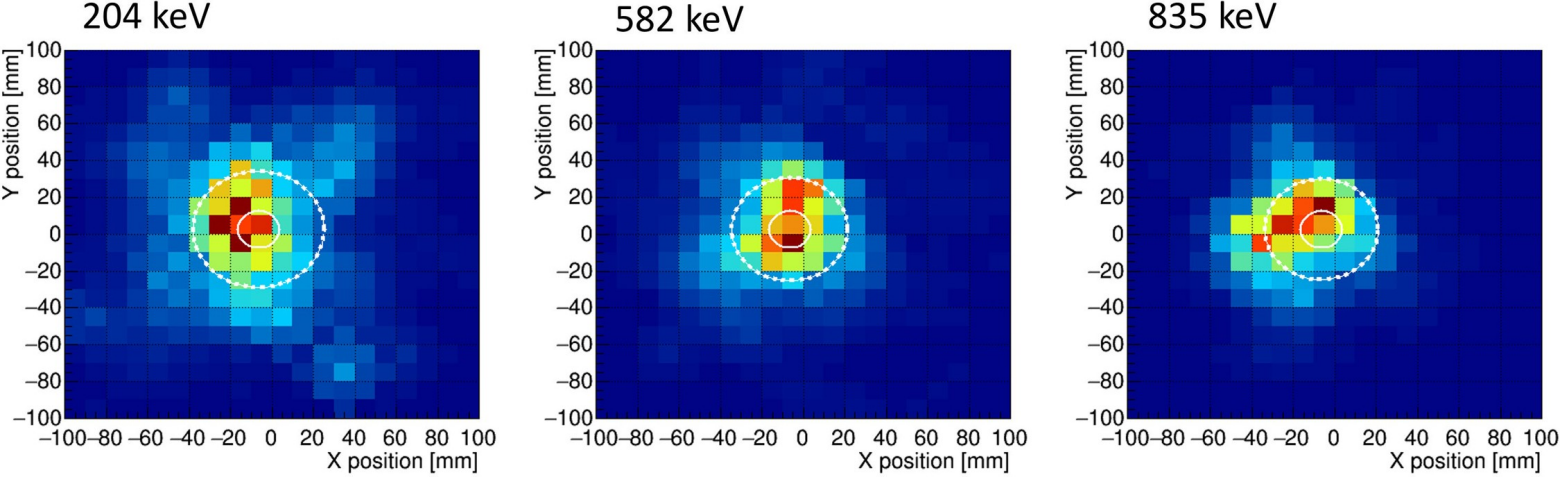
Compton camera images obtained from ^95m^Tc contained in a 10-mm-diameter vial. Energy windows are set at (a) 204 keV, (b) 582 keV, and (c)835 keV. These images were reconstructed using an interactive reconstruction technique. The solid line shows a vial size of 10 mm in diameter. Fifty percent of all events exist inside of the dashed line circle.

**Fig 8:**
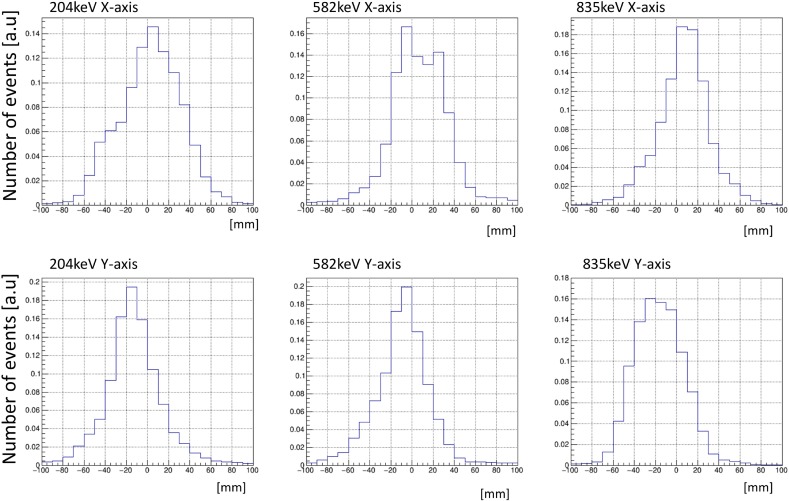
The histograms of sliced images of Fig 7 are shown. The upper three panels are sliced within ±15 mm along the X-axis. The energies of these histograms are 204, 582, and 835 keV, respectively, from left to right. The lower panels are sliced within ±15 mm belongs along the Y-axis and each energy is same as those on the X-axis.

The white circles in Fig 7 show the position of the vial containing the ^95m^Tc solution. The point spread function of ETCC is limited by the uncertainty of the initial moment of the Compton-recoil electron and multiple scattering of the Compton-recoil electron in the gas. The uncertainty of the initial electron moment appears as a Lorentz distribution [32], and multiple scattering occurs as a Gaussian-like distribution [33]. The histograms of sliced images of Fig 7 are shown in Fig 8. The upper three panels are sliced within ±15 mm along the X-axis. The energies of these histograms are 204, 582, and 835 keV, respectively, from left to right. The lower panels are sliced within ±15 mm along the Y-axis, and each energy is same as those on the X-axis. In such a complicated case, circles in which 50 or 68% of the events contained are often used as the definition of the angular resolution (for example, see references [34, 35]). In the present study, to evaluate the relative spatial resolutions of the three images, we also plotted dotted line circles within which 50% of all events exist. The diameters of the dotted line circles were approximately 49.3 mm, 50.5 mm, and 56.7 mm for 835 keV, 582 keV, and 204 keV, respectively. This result shows that the spatial resolution of ETCC mapping depends on the γ-ray energy. The diameter of the dotted line circles decreased with increasing γ-ray energy. This trend is consistent with the energy resolution dependence that the energy resolution of the measured γ-rays increases with an increase in the γ-ray energy. In our previous study [24], γ-rays at 204 keV from ^95m^Tc were measured by an ETCC, in which most γ-rays are located in a circle of a diameter with 30 mm. However, we did not estimate the circle within which 50% of all events exist in the previous study; thus, we cannot directly compare the present result at 204 keV with the previous one. Again we noted that the energy resolution at high energies of 582 keV and 835 keV is higher than that at 204 keV, corresponding to previous studies [36, 37].

In the present case, the background level strongly affects the diameter of the dotted line circle. As shown in Fig 6, the background level in the low-energy region is higher than that in the high-energy region. The ETCC system cannot measure the total energy in all cases. Because Compton scattering in the second γ-ray detector may occur, high-energy γ-rays increase the background level in the low-energy region. The present results show that the ETCC system can measure the images for different γ-ray energies even if γ-ray tracers emit various energy γ-rays and that the image in the high-energy region is clearer than that in the low-energy region.

## Conclusion

In the present study, we produced ^95m^Tc using the ^95^Mo(p, n)^95m^Tc reaction to measure images of ^95m^Tc using an ETCC. After chemical separation, 500 kBq of ^95m^Tc was obtained. The recycling procedure for the ^95^MoO_3_ isotope-enriched target material was examined, and the recovery yields of ^95^Mo were 70%-90%. The ETCC images were obtained for γ-rays with three different energies, 204 keV, 582 keV, and 835 keV, emitted from ^95m^Tc. The spatial resolution increased as the γ-ray energy increased, indicating that the ETCC system can measure images even in the presence of γ-rays with various energies.
